# A Modified Skip-Gram Algorithm for Extracting Drug-Drug Interactions from AERS Reports

**DOI:** 10.1155/2020/1747413

**Published:** 2020-04-13

**Authors:** Li Wang, Wenjie Pan, QingHua Wang, Heming Bai, Wei Liu, Lei Jiang, Yuanpeng Zhang

**Affiliations:** ^1^Department of Medical Informatics, Medical School, Nantong University, Nantong 226001, China; ^2^Research Center for Intelligence Information Technology, Nantong University, Nantong 226001, China; ^3^Department of Rheumatology and Immunology, Changzheng Hospital, The Second Military Medical University, Shanghai 200433, China

## Abstract

Drug-drug interactions (DDIs) are one of the indispensable factors leading to adverse event reactions. Considering the unique structure of AERS (Food and Drug Administration Adverse Event Reporting System (FDA AERS)) reports, we changed the scope of the window value in the original skip-gram algorithm, then propose a language concept representation model and extract features of drug name and reaction information from large-scale AERS reports. The validation of our scheme was tested and verified by comparing with vectors originated from the cooccurrence matrix in tenfold cross-validation. In the verification of description enrichment of the DrugBank DDI database, accuracy was calculated for measurement. The average area under the receiver operating characteristic curve of logistic regression classifiers based on the proposed language model is 6% higher than that of the cooccurrence matrix. At the same time, the average accuracy in five severe adverse event classes is 88%. These results indicate that our language model can be useful for extracting drug and reaction features from large-scale AERS reports.

## 1. Introduction

Drug-drug interactions (DDIs) accounted for over 30% of all adverse drug events [1]. More serious fact is that large quantity of DDIs manifested after a long period of exposure. As a result, AERS reports have been served as the cornerstone for detecting unanticipated interactions. The development of computational prediction and assessment of DDIs become attractive to the US FDA and pharmaceutical companies [2]. Harpaz et al. developed a taxonomy that characterized the associations and predicted several potential multi-item drug adverse effects [3]. They revealed that duplicate reports caused spurious associations. Tatonetti et al. constructed a drug-reaction frequency matrix and used Fisher's exact test for feature extraction from frequency matrices for DDI prediction [4]. Logistic regression was used for classification. Predicted DDIs were significantly enriched for known effects. Cheng and Zhao integrated drug phenotypic, therapeutic, chemical, and genomic properties to predict DDIs [5]. These four types of drug-drug similarities were calculated as features of each drug-drug pair for prediction. Five machine learning algorithms were implemented, and they found that integration of multidata sources can improve the performance of DDI prediction. Cami et al. proposed a Predictive Pharmacointeraction Network [6]. They exploited the network structure of all known DDIs, combined with various taxonomic and intrinsic properties of drugs to predict unknown DDIs. While these methods performed well, their limitations are obvious either. From the above, similarity-based methods rely on various profiles including drug molecular structure profiles, drug-drug interaction profiles, and pharmacophoric profiles [7, 8]. First of all, barely any of the previous work took a systematic data preprocessing method before taking advantage of AERS reports; a standard language description framework should be used to organize all the reports. Second, in the face of the large amount of free text reports, to solve the problem that the integration of profiles cost large amount of manually check and selection biases, a language concept representation model is urgently needed. Third, the existing approaches do not seem to extract features from AERS reports efficiently and to test the quality of the new-mined DDI concept by our MSG model, which also are needed to be distinguished in the DrugBank database.

The skip-gram algorithm was one of the language models set in the open-source word2vec [9, 10]. This algorithm was used to render distributional representation of words from large-scale unmarked text. The skip-gram algorithm has been widely acknowledged and successfully applied to many natural language processing tasks, such as text clustering, entity completion in incomplete knowledge bases or ontologies, and text retrieval [11–13]. At the same time, there are few researchers focusing on applying this language model to mining the pharmacovigilance information from large-scale reports in free text format.

The main contribution of this work can be listed as follows:

1. We proposed a new language concept representation model by changing the scope of the window value in the original skip-gram algorithm
2. Compared to the previous traditional language model, the new model can extract features of drug name and reaction information from large-scale AERS reports more efficiently
3. The new drug-drug interaction datasets can be mined through the use of the proposed MSG language concept representation model

## 2. Method

**Figure 1:**
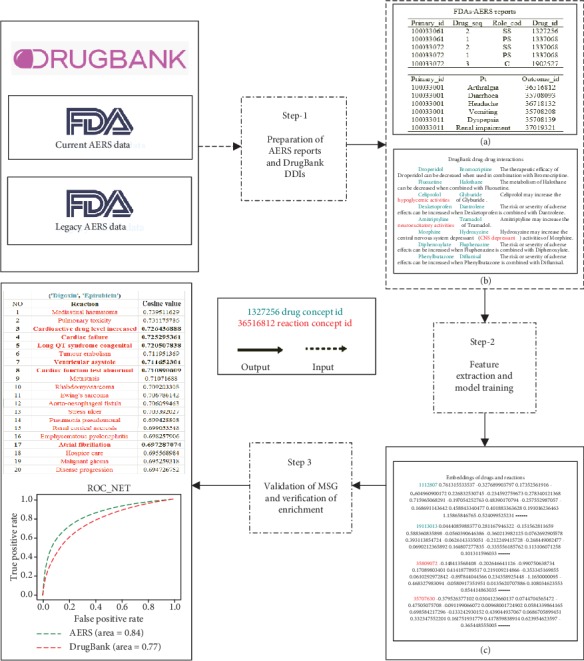
The scheme of DDI extraction based on the MSG algorithm.

As shown in Figure 1, in the whole research scheme, we proposed a modified skip-gram (MSG) algorithm for drug name and reaction description feature extraction from FDA AERS reports, and the description of DDIs in DrugBank was enriched.

In Step 1, original free text AERS reports are transformed into structured tables (Figure 1(a)). Our study refers to the framework of OHDSI (Observational Health Data Sciences and Informatics) and puts Banda et al.'s research into practice, completing the cleaning and standardization of AERS reports [14, 15]; all the structured tables are stored in a PostgreSQL database. In addition, we extracted DrugBank DDI and toxicity data into text files as shown in Figure 1(b).

In Step 2, the MSG algorithm was applied to calculate the embeddings of drug name and reaction descriptions from AERS reports and DrugBank DDI. The vectors with a dimension of 100 were represented as features of drug name and reaction description. All the names of drugs and reactions are converted from string to the numbers as shown in Figure 1(c). For example, one drug name is represented as a drug concept ID 1327356 with 100 dimension separated numbers.

In Step 3, a logistic regression classifier was used to validate the above embedding values. To compare the quality of embeddings generated from MSG, CM-TF-IDF, another traditional language representation model, was also tested. We chose to assess the performance of the classifier in comparing the area under the curve (AUROC) of AERS reports and DrugBank DDI with a cross-validation approach.

Finally, the descriptions of DDIs in five severe adverse event classes were enriched into the current adverse event results.

### 2.1. Data Collection and Preprocess

We collected AERS reports from the FDA's website between 2004 and 2014. In addition, SIDER was used as the gold standard for positive reference samples [16]. DDI data and drug toxicity data were extracted from the DrugBank database [17].

Although it is a free and publicly available resource, the FDA AERS data still presents multiple hurdles in consolidating all relevant data. To avoid producing unreliable and irreproducible results, widely accepted data preprocessing methods were referred to and put into practice. Thanks to the efforts of large communities such as the Observational Health Data Sciences and Informatics (OHDSI) [14], we can focus more on model building than on lots of time-wasted efforts such as cleaning and standardizing the AERS reports. For details of preprocessing FDA AERS reports, we referred to Banda et al.'s research [15]. First, AERS reports in Extensible Markup Language (XML) format were extracted into seven individual tables; these tables were loaded onto PostgreSQL. Second, a demo table was created for missing value imputation and case deduplication. Missing value imputation was performed on four demographic fields (age, sex, country, and event date). As a case may exist in the legacy AERS dataset or in the new FAERS dataset, different unique row keys were managed in a case deduplication step. Finally, regular expression was taken as the main method for mapping drug and reaction concept into the OHDSI standard vocabulary concept identifier (consisting of RxNorm CUIs and MedDRA standard codes).

After the preprocessing of AERS reports, in total, 4,493,179 reports are achieved, within which 713,441 reports listed exactly two drugs. To ensure reasonable estimates and statistical significance, at least ten AERS reports are required to support one given drug concept [18]. We selected ten as the threshold to filter out drug concepts in AERS reports. As a result, 675 drug concepts are filtered out and existed in DrugBank DDI dataset drug concepts either. These 675 drugs are included in Reference Drug Lists (RDL).

### 2.2. Modified Skip-Gram (MSG) Model

The original skip-gram algorithm was modified for drug name and reaction description feature extraction from FDA AERS reports and DrugBank DDIs. Based on distributional hypothesis theory [9], a word can be characterized into an embedding value by contexts, which are the surrounding words around its position in the sentence. These embeddings encode the semantic meanings of the target word into a low-dimensional vector. In this research, all the drugs with reaction words are encoded into a low-dimensional vector. Our modified skip-gram model was trained by the hierarchical softmax procedure presented in Mikolov et al.'s research [10]. The objective of the skip-gram model is to maximize the log probability:

(1) $$\begin{array}{c} \underset{\left(w,c\right)\in D}{\sum }\underset{w_{j}\in c}{\sum }\log P\left(w\;|\;w_{j}\right). \end{array}$$

In Equation (1), *c* is the limited set of drugs and reactions in one AERS report. When *w* denotes the drug, *w*_*j*_ denotes the reaction in the report and vice versa. In the equation above, *P*(*w* | *w*_*j*_) can be detailed as follows:

(2) $$\begin{array}{c} P\left(w\;|\;w_{j}\right)=\frac{\exp\left(e^{\prime }{\left(w\right)}^{T}e\left(w_{j}\right)\right)}{\sum_{w^{\prime }\in V}\exp\left(e^{\prime }{\left(w^{\prime }\right)}^{T}e\left(w_{j}\right)\right)}. \end{array}$$

In Equation (2), *e*′(*w*) is the embedding of drug *w* (reaction either), *w*′ is one of the words in the vocabulary *V* consisting of drug name and reaction descriptions.

**Figure 2:**
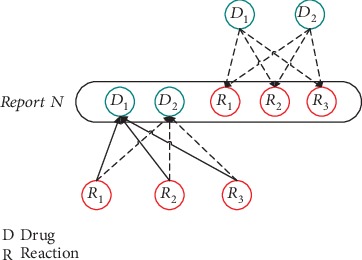
Dynamic scope of the window of the modified skip-gram model.

The key difference between the original skip-gram and modified skip-gram is the way we define “context.” In the original skip-gram, the context is 2*n* words around the current target word. The 2*n* words are composed of *n* words forward—the current target word—and *n* words backward. In default, *n* is set in five. According to the particularity orders of words in drug-drug interaction reports, the context of each drug name word is every reaction description word appearing in the sentences of each AERS report in our modified skip-gram. When it comes to reaction description words, the context words *c* are changed into every drug name word in the corresponding sentences. As shown in Figure 2, the contexts of Drug_1_ are Reaction_1_, Reaction_2_, and Reaction_3_.

### 2.3. Cooccurrence Matrix Based on Term Frequency-Inverse Document Frequency (CM-TF-IDF)

Term frequency-inverse document frequency (TF-IDF) is well known as a statistical method for evaluating the importance of one word in the corpus [19]. The importance of the word is increased in direct proportion to how many times it appears in the file and at the same time is declined in inverse proportion to how many times it appears in the whole corpus. In Equation (3), *n*_*i*,*j*_ is the time word *t*_*i*_ appearing in the file *d*_*j*_ and ∑_*k*_*n*_*k*,*j*_ is the sum of frequencies of all words appearing in the file *d*_*j*_. |*D*| denotes the total number of documents, and |{*j* : *t*_*i*_ ∈ *d*_*j*_}| is the number of documents which contain the word *t*_*i*_ in the corpus.

(3) $$\begin{array}{c} {\text{tfidf}}_{i,j}=\frac{n_{i,j}}{\sum_{k}n_{k,j}}\times \log\frac{\left|D\right|}{\left|\left\{j:t_{i}\epsilon d_{j}\right\}\right|}. \end{array}$$

**Figure 3:**
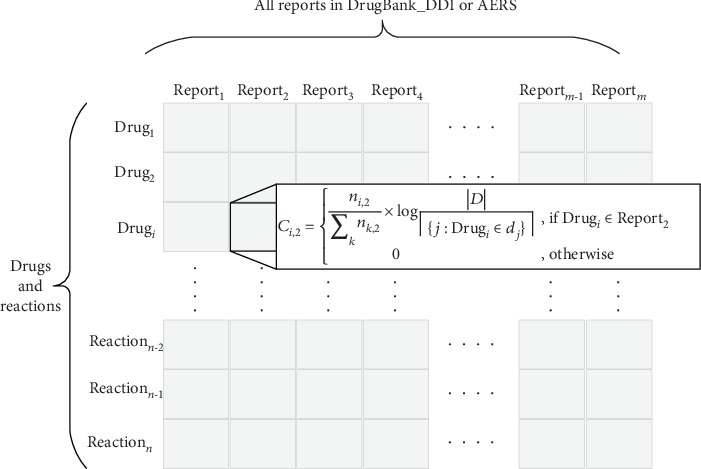
Drug/reaction report cooccurrence matrix based on tfidf.

As shown in Figure 3, we constructed a drug name/reaction description report cooccurrence matrix based on TF-IDF for feature vectorization of drugs and reactions. For example, if Drug*_i_* was recorded in Report_2_, the element in the matrix is the tfidf of Drug*_i_*; otherwise, the element is zero.

### 2.4. Logistic Regression

According to the MSG algorithm, drug encoded its reaction information into a low-dimensional vector after the MSG training. CM-TF-IDF also generated drug and reaction vectors from the cooccurrence matrix. These low-dimensional vectors are rendered as features for identifying whether or not the drug pairs are associated with the adverse event class. As logistic regression has been widely used in pharmacovigilance and achieved good performance, it was applied in our research [4–6].

**Figure 4:**
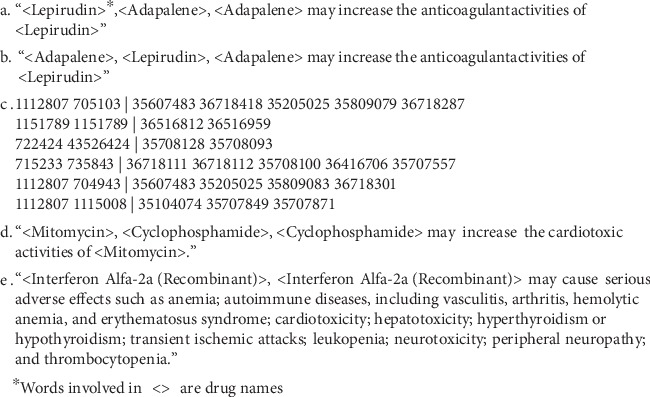
Examples of DDIs in DrugBank and report in DrugBank_Toxicity.

Referring to distinct severe adverse event classes presented by Tatonetti et al. [4], five clinically significant adverse event classes are taken into consideration for binary classification and DDI enrichment: Renal Impairment (REI), Hepatotoxic (HTT), Abnormal Blood Pressure (ABP), Cardiotoxicity (CDT), and Neurotoxic (NET). The logistic regression model requires positive and negative labels which indicate whether or not the pair of drugs is associated with the adverse event class. Because there is no well-recognized gold standard for drug-drug interaction, we crosssearched three datasets (DrugBank_Toxicity, DrugBank_DDI, and SIDER) and compiled three strategies as follows to define the positive reference samples, see Figure 4.

In the first strategy, if at least one of the drugs in one pair existed in SIDER's specific drug lists where drugs are associated with the adverse event, we labeled this pair of drugs as positive.

In the second strategy, if at least one of the drugs in one pair manifests as an adverse event-associated toxicity in DrugBank_Toxicity, we labeled this drug pair as positive.

In the third strategy, according to DrugBank_DDI, if the pair is known to interact which results in the adverse event, the pair is labeled as positive.

## 3. Evaluation and Experiment Results

To assess the performance of the scheme based on MSG, we compared vectors generated from MSG with CM-TF-IDF. The receiver operating characteristic (ROC) is used for the evaluation of binary classifiers [20]. To obtain robust estimates, we performed 10-fold cross-validation; the whole dataset was divided into ten cross-validation splits. During each cross-validation step, a set of nine cross-validation splits was used for model training while the tenth sample set was applied as the test set.

No matter how the embedding was generated, all embedding models are constructed based on the distributional hypothesis. That is to say, if two words have similar context, their value of embeddings is close in the low-dimensional space. Furthermore, the value of drug pair embeddings is theoretically close with its interactions in the low-dimensional space. As a result, we extended this idea to the enrichment of DDIs in DrugBank. Cosine between drug pair embeddings and reaction embeddings was calculated as the reference for ranking.

Although the drug and reaction embeddings were generated after the MSG training, there are no explicit drug pair embeddings. As shown in Equation (3), we empirically constructed drug pair embeddings by addition. For details about variables in Equation (3), *e*_Drug1_ = (*a*_1_, *a*_2_, ⋯, *a*_*n*_) and *e*_Drug2_ = (*b*_1_, *b*_2_, ⋯, *b*_*n*_). *a*_*i*_ and *b*_*i*_ are the values of each *n* dimension of drug embedding. Cosine between drug pair embedding and reaction embedding was calculated according to Equation (4). In Equation (4), **e**_Reaction_ = (*r*_1_, *r*_2_, ⋯, *r*_*n*_). *r*_*i*_ is the value of each *n* dimension of reaction embedding.

(4) $$\begin{array}{c} e_{\left(\text{Drug}1,\text{Drug}2\right)}=\left(a_{1}+b_{1},a_{2}+b_{2},\cdots ,a_{n}+b_{n}\right), \end{array}$$

(5) $$\begin{array}{c} \cos\left(e_{\left(\text{Drug}1,\text{Drug}2\right)},e_{\text{Reaction}}\right)=\frac{\sum_{1}^{n}\left[\left(a_{i}+b_{i}\right)\times r_{i}\right]}{\sqrt{\sum_{1}^{n}{\left(a_{i}+b_{i}\right)}^{2}}\times \sqrt{\sum_{1}^{n}{r_{i}}^{2}}}. \end{array}$$

In summary, we sorted cosine of candidate reactions and drug pairs. Finally, top 20 candidate reactions were used to enrich descriptions of DDIs in DrugBank.

In total, 713,441 reports listed only two drugs in FDA AERS reports and 561,180 DDIs in the DrugBank database. We only included the record where drug pairs are listed in RDL. It is worth noting that deduplication of DDIs in DrugBank is also important. As shown in Figures 3(a) and 3(b), there are two DDIs from DrugBank. These two DDIs actually represent the same knowledge, so only one of them was kept for our research. As a result, 218,866 AERS reports and 46,203 DrugBank DDIs were included in our analysis. As shown in Figure 3(c), 218,866 AERS reports were exported from PostgreSQL into plain text format for MSG training. On the left side of the symbol “|” are drug concept ID and right side of the symbol “|” are reaction concept ID. Four crucial parameters of the MSG model are shown in Table 1. “Min count for drugs or reactions” was set to 10 as described in Section 3. “Starting alpha” and “Dimensionality of word embeddings” were set to default as 0.025 and 100, separately. “Gradient calculation” was set to Hierarchical softmax for performance improvement.

As mentioned in Section 3, five logistic regression models required five sets of samples which consist of positive and negative labels. The detailed distribution of positive samples in three datasets (DrugBank_DDI, DrugBank_Toxicity, and SIDER) is listed in Table 2. In column DrugBank_Toxicity and SIDER, the number is the positive samples of drugs. In column DrugBank_DDI, the number is the positive samples of drug pairs. For example, as shown in Figure 3(d), the DDIs have the keyword “cardiotoxic”. As a result, the drug pair <Mitomycin, Cyclophosphamide> was one of the 544 positive samples (Table 2) in Cardiotoxicity (CDT) adverse event class. In DrugBank_Toxicity dataset, as shown in Figure 3(e), the report of drug <Interferon Alfa-2a (Recombinant)> has the keyword “cardiotoxicity”; we included the drug pair as a positive sample in Cardiotoxicity (CDT) adverse event class if the drug pair has drug <Interferon Alfa-2a (Recombinant)>. In the SIDER dataset, we marked the drug pair as a positive sample in Cardiotoxicity (CDT) adverse event class if the drug pair has the drug listed in 448 manually checked drugs (Table 2).

### 3.1. Validation of Logistic Regression Models Based on MSG and CM-TF-IDF

**Figure 5:**
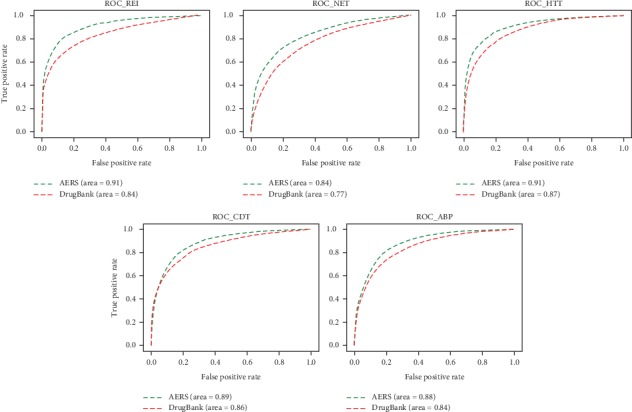
ROC of ten logistic regression models based on MSG.

**Figure 6:**
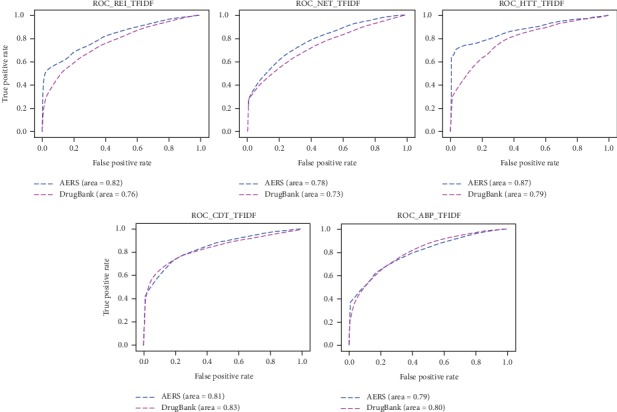
ROC of ten logistic regression models based on CM-TF-IDF.

**Figure 7:**
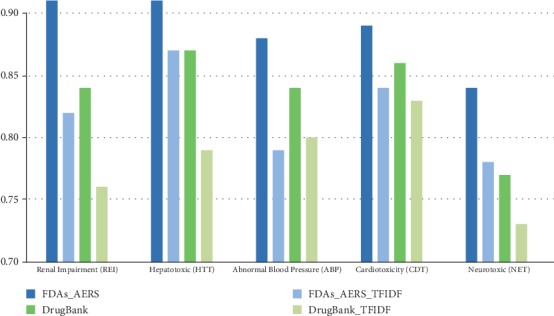
AUROC of twenty logistic regression models.

We trained and validated logistic regression models for Renal Impairment (REI), Hepatotoxic (HTT), Abnormal Blood Pressure (ABP), Cardiotoxicity (CDT), and Neurotoxic (NET). When it comes to the vectors generated from MSG, each one of the five clinical significant adverse event classes has two logistic regression models based on AERS reports and the DrugBank DDI dataset individually. When it comes to the vectors generated from CM-TF-IDF, ten logistic regression models are also trained like MSG. In order to avoid dimension disaster of CM-TF-IDF, principal component analysis (PCA) was used for feature dimensionality reduction of CM-TF-IDF. The ROC curve of five adverse event classes based on MSG and CM-TF-IDF is shown in Figures 5 and 6; AUROC of five adverse event classes is shown in Figure 7. As shown in Figure 7, five logistic regression models based on AERS reports achieved a higher value of AUROC than five logistic regression models based on DrugBank DDIs. All AUROC based on MSG in Figure 7 are higher than those based on CM-TF-IDF, which means that our modified skip-gram model can extract features from AERS reports and DrugBank DDI dataset more effectively than the traditional statistical method CM-TF-IDF. At the same time, logistic regression has a good performance of classification in these five adverse event classes as we expected.

### 3.2. Enrichment of DDIs in DrugBank

We calculated the cosine of 1,650 DrugBank DDIs for description enrichment and taken MedDRA for verification of description enrichment in five adverse event classes: Renal Impairment (REI), Hepatotoxic (HTT), Abnormal Blood Pressure (ABP), Cardiotoxicity (CDT), and Neurotoxic (NET).

As we know, there are five levels in the MedDRA hierarchy, arranged from specific to general: {System Organ Class (SOC)}, {High level Group Terms (HLGT)}, {High Level Terms (HLT)}, {Preferred Term (PT)}, and {Lowest Level Term (LLT)} [19]. In order to verify the enrichment of DDIs in DrugBank, twenty-seven {System Organ Class (SOC)} are taken into our consideration. Taken Neurotoxic (NET) as example, when we verified the enrichment of DDIs in Neurotoxic (NET), {Nervous system disorders} in {System Organ Class (SOC)} is set as gold standard for the right reactions in Neurotoxic (NET). If at least one of the reactions in top 20 of the drug pair is under the {Nervous system disorders} category, then we define the description enrichment of the drug pair in Neurotoxic (NET) is valid. For example, the enrichment of drug pair <Digoxin, Epirubicin> in Cardiotoxicity (CDT) class is shown in Table 3. Six bold font reactions are verified under the {Cardiac disorders} in System Organ Class (SOC), so the description enrichment of drug pair <Digoxin, Epirubicin> is valid.  Table 4 shows the details of DDI enrichment of five classes. In total, 1,456 description enrichments are verified valid, and the average accuracy is 0.882424, which means the description of DDIs in DrugBank is enriched efficiently by using MSG model.

## 4. Discussion

In order to verify and demonstrate the advantage of our presented new scheme, we repeated the whole experiments using the cooccurrence matrix based on tfidf model to generate drug and adverse feature vectors. From the results of ten logistic regression models (as shown in Figure 7 the results show that five logistic regression models based on AERS reports all achieved higher value of AUROC than five logistic regression models based on DrugBank DDIs. In FDA AERS datasets, the vectors generated by the MSG can give better performance in feature extraction than by the tfidf-based cooccurrence matrix model. The main reasons behind the above results are as follows: (1) the cooccurrence matrix based on tfidf model can cause dimensionality disaster when the data size is large; some features are bound to be lost when using the principal component analysis (PCA). The MSG model defines the dimension of the space vector at initialization, which avoids the work of secondary feature engineering and avoids the loss of feature information. (2) The MSG model constructs a Huffman tree based on word frequency during initialization, and the activation function of each node is softmax, which greatly shortens the time for updating weights and vectors in the whole learning process. Because of these reasons, the MSG model can be applied to large-scale datasets compared with the traditional tfidf-based cooccurrence matrix and also can quickly perform feature learning. At the same time, we also found that the MSG model can perform well for the noisy dataset. When MSG model is applied in DrugBank data, all the noisy data are not specifically cleaned after the alignment of drug and adverse reaction strings. However, from the five AUROC values (as shown in Figure 7 DrugB_MSG), the average AUROC values of the five major adverse reaction groups are around 0.8, which shows that the MSG model can also effectively generate feature vectors from the noise dataset.

## 5. Conclusions

In this work, we proposed an efficient method of feature vector extraction and calculation from FDA AERS and DrugBank texts based on the modified skip-gram model. Feature vectors are taken to expand drug-drug interaction datasets of the DrugBank database. All the accuracy values are higher than 80% (as shown in Table 4) and show that these new features are valuable in five severe adverse event classes. The contribution of clinicians may accelerate the process of MSG model application in the clinical field.

In the future, on the one hand, we will continue to optimize the accuracy of the word vector and try to integrate the attention mechanism into the language representation algorithm, and on the other hand, we are going to apply the detection of adverse drug reactions to the actual electronic medical record medication prescription system, so as to promptly remind doctors and patients when using drugs.

## Figures and Tables

**Table 1:** Parameters of the modified skip-gram model.

	Dimensionality of word embeddings	Starting alpha	Min count for drugs or reactions	Gradient calculation
Parameters	100	0.025	10	Hierarchical softmax

**Table 2:** Positive reference samples of five event classes.

Event class	DrugBank_DDI	DrugBank_Toxicity	SIDER
Renal Impairment (REI)	117	47	270
Hepatotoxic (HTT)	11	29	265
Abnormal Blood Pressure (ABP)	757	132	275
Cardiotoxicity (CDT)	544	51	448
Neurotoxic (NET)	221	158	298

**Table 3:** Enrichment of drug pair <Digoxin, Epirubicin> in DrugBank.

No.	Reaction	Cosine
1	Mediastinal haematoma	0.739511629
2	Pulmonary toxicity	0.731175786
**3**	**Cardioactive drug level increased**	**0.726436888**
**4**	**Cardiac failure**	**0.725295361**
**5**	**Long QT syndrome congenital**	**0.720507838**
6	Tumour embolism	0.711951369
**7**	**Ventricular asystole**	**0.711652301**
**8**	**Cardiac function test abnormal**	**0.710890609**
9	Metastasis	0.71071688
10	Rhabdomyosarcoma	0.709203308
11	Ewing's sarcoma	0.706786142
12	Aorto-oesophageal fistula	0.706059463
13	Stress ulcer	0.703392027
14	Pneumonia pseudomonal	0.699428808
15	Renal cortical necrosis	0.699053548
16	Emphysematous pyelonephritis	0.698257906
**17**	**Atrial fibrillation**	**0.697287074**
18	Hospice care	0.695568984
19	Malignant glioma	0.695259318
20	Disease progression	0.694726752

**Table 4:** Details of drug pair DDI enrichment in DrugBank.

Event class	Number of DDIs	Number of valid enrichments	Accuracy
Renal Impairment (REI)	117	99	0.846154
Hepatotoxic (HTT)	11	9	0.818182
Abnormal Blood Pressure (ABP)	757	660	0.871863
Cardiotoxicity (CDT)	544	494	0.908088
Neurotoxic (NET)	221	194	0.877828
Total	1650	1456	0.882424

## Data Availability

We do not want to share our data due to our furture works.
